# Supplementary material for: Physical Therapy Utilization and Morbidity Outcomes After Breast Cancer Surgery: A Longitudinal Analysis of Three Combined Cohorts
Source: Cancers (Basel). 2025 Oct 11;17(20):3296. doi: 10.3390/cancers17203296 (PMC12562795; doi:10.3390/cancers17203296)
Supplement: Supplementary file 1 [file cancers-17-03296-s001.zip › cancers-3888799-Supplementary File S1.pdf]

Supplementary File S1. ARM-BCT Instrument and Scoring.

Overview. The Arm Morbidity following Breast Cancer Treatments (ARM-BCT) is a 17-item screening instrument to estimate risk of future upper-extremity morbidity after breast-cancer surgery. Items combine patient- and treatment-related factors into a single score (range 1–20; higher = greater risk).

Scoring and risk tiers. Total score = sum of item points (see coding table). Using validated cut-points, risk is classified as: Low <6; High >7. In the present study, when available, ARM-BCT tier was included as a categorical covariate in multivariable models of PT uptake/timing.

Missing data. The instrument permits scoring with incomplete responses: a total ARM-BCT score is computed when ≥8 of 17 items are completed. If fewer than 8 items are available, the ARM-BCT total and risk tier are set to NA and excluded from tier-adjusted analyses (available-case approach). No prorating was applied; totals reflect the sum of completed item points, and risk tiers were assigned based on that total.

ARM-BCT Questionnaire

Instructions: Please answer the following questions. Responses are coded for scoring according to the rules below.

Section 1 – Age

- 0 = 18–56 years
- 1 = 57–120 years

Section 2 – Body Mass Index (BMI)

- Calculate BMI = weight (kg) / [height (m)]^2 (calculation done separately, not shown on questionnaire).
- 0 = BMI < 28
- 1 = BMI ≥ 28

Section 3 – Comorbidities

- 0 = No comorbidities
- 1 = Neurological/orthopedic limitations, shoulder surgery/trauma (same side), fibromyalgia, or chronic pain

Section 4 – Type of Surgery

- 1 = Lumpectomy
- 2 = Mastectomy (partial or total)

Section 5 – Number of Lymph Nodes Removed

- 0 = None
- 1 = 1–4 nodes
- 2 = 5–12 nodes
- 3 = ≥13 nodes

Section 6 – Breast Reconstruction

- 0 = No
- 1 = Yes

Section 7 – Postoperative Complications

- 0 = No
- 1 = Yes (bleeding, infection, reoperation, seroma drainage)

Section 8 – Cancer Stage

- 0 = No disease
- 1 = Pre-invasive / localized
- 2 = Advanced / metastatic

Section 9 – Radiation Therapy

- 0 = No
- 1 = Yes

Section 10 – Chemotherapy

- 0 = No
- 1 = Yes (received or planned)

Section 11 – Social/Family Support

- 0 = Yes
- 1 = No

Section 12 – Sleep Disturbance After Surgery

- 0 = No
- 1 = Yes

Section 13 – Mood in Past Week (0=positive, 10=very depressed)

- Patient enters a number 0–10
- 0 = <3.5
- 1 = ≥4

Section 14 – Anxiety in Past Week (0=none, 10=severe)

- Patient enters a number 0–10
- 0 = <3.5
- 1 = ≥4

Section 15 – Severe Pain During Hospitalization (Numeric pain rating scale>6/10)

- 0 = No
- 1 = Yes

Section 16 – Physiotherapy Instruction During Hospitalization

- 0 = Yes
- 1 = No

Section 17 – Physical Activity Level

- Patient reports number of sessions per week in each category (≥30 min/session):
- Low intensity (Feldenkrais, light walking, slow swimming) → multiply ×1

- Moderate intensity (fast walking, yoga, Pilates, light cycling, tennis) → multiply ×2
- High intensity (running, football/basketball, functional training, CrossFit) → multiply ×3
- Scoring rule:
- ≤2 = Sedentary → code = 1
- 3–6 = Moderately active → code = 0
- ≥7 = Active → code = **–1 (protective factor)**

Scoring System

• A higher score = higher morbidity risk.

• Final cut-off:

- Score ≥7 → High Risk
- Score <7 → Low Risk

Abbreviations. ALND, axillary lymph-node dissection; BMI, body mass index; PT, physiotherapy; NRS, numeric rating scale.
