# Supplementary material for: Physical Therapy Utilization and Morbidity Outcomes After Breast Cancer Surgery: A Longitudinal Analysis of Three Combined Cohorts
Source: Cancers (Basel). 2025 Oct 11;17(20):3296. doi: 10.3390/cancers17203296 (PMC12562795; doi:10.3390/cancers17203296)
Supplement: Supplementary file 1 [file cancers-17-03296-s001.zip › cancers-3888799-Supplementary Table S1.pdf]

Supplementary Table S1. Clinical predictors of postoperative physical therapy utilization.

Predictors	Adjusted OR	95% CI	p value
Time window	0.76	0.63–0.91	0.003
Comorbidity	1.38	0.87–2.17	0.170
Lumpectomy	2.8	0.82–9.52	0.097
Mastectomy	2.1	1.08-4.26	0.028
Prophylactic mastectomy	1.7	0.83–3.53	0.142
Axillary lymph-node dissection	2.64	1.50–4.64	0.001
Breast reconstruction	1.95	0.87–4.38	0.105
Decreased function	1.33	0.76–2.34	0.321
QuickDASH	0.98	0.95–1.02	0.331
ROM limitation	1.48	0.88–2.49	0.138
Lymphedema	3.06	1.42–6.57	0.004
Pain level	0.87	0.79–0.96	0.007
PT during hospital stay	1.36	1.00–1.64	0.048
Complementary treatments	2.82	1.82–4.38	< 0.001

Abbreviations. OR, odds ratio; CI, confidence interval; PT, physiotherapy; ROM, range of motion; ALND, axillary lymph-node dissection (≥5 nodes); QuickDASH, Disabilities of the Arm, Shoulder and Hand (0–100; higher = greater disability). Note. Outcome = receipt of postoperative PT (1 = yes, 0 = no). Odds ratios are adjusted for all covariates listed. Binary factors are coded yes vs no (e.g., comorbidity, lymphedema, PT during hospital stay, complementary treatments). Time window is modeled ordinally per 6-month increment from 0–6 to 25–36 months (higher values = longer interval).

Continuous predictors (QuickDASH and Pain) enter the model per 1-point increase. “Surgery type” appears as indicator variables with an implicit reference category (not shown). Complementary treatments include any self-reported shiatsu, reflexology, yoga/Pilates-based rehabilitation, or acupuncture after surgery. *p* values are two-tailed; $p < 0.05$ denotes statistical significance.
